# Tumor Colonization and Therapy by *Escherichia coli* Nissle 1917 Strain in Syngeneic Tumor-Bearing Mice Is Strongly Affected by the Gut Microbiome

**DOI:** 10.3390/cancers14246033

**Published:** 2022-12-07

**Authors:** Ivaylo Gentschev, Ivan Petrov, Mingyu Ye, Lina Kafuri Cifuentes, Romy Toews, Alexander Cecil, Tobias A. Oelschaeger, Aladar A. Szalay

**Affiliations:** 1 Department of Biochemistry/Cancer Therapy Research Center (CTRC), Theodor-Boveri-Institute, University of Wuerzburg, 97074 Wuerzburg, Germany; 2 Helmholtz Zentrum München, German Research Center for Environmental Health (GmbH), Research Unit of Molecular Endocrinology and Metabolism, 85764 Neuherberg, Germany; 3 Institut für Molekulare Infektionsbiologie, D-97080 Würzburg, Germany; 4 Department of Radiation Oncology, Rebecca & John Moores Comprehensive Cancer Center, University of California, San Diego, CA 92093, USA; 5 Department of Pathology, Center of Immune Technologies, Stanford University School of Medicine, Stanford, CA 94305, USA

**Keywords:** *E. coli* Nissle 1917, probiotic bacterium, gut microbiome, bacterial cancer therapy, tumor, syngeneic tumor-bearing mice, tumor colonization

## Abstract

**Simple Summary:**

Cancer is a leading cause of disease-related death in humans and in domestic animals. Despite recent progress in the diagnosis and treatment of advanced cancer, the overall patient treatment outcome did not substantially improve. Bacterium-mediated cancer therapy is one promising method of cancer treatment. The most important challenges for the successful clinical use of bacteria are ensuring their preferential tumor colonization and initiating functional tumor-specific immunity. In the current work, we describe the influence of the gut microbiome on *Escherichia coli* Nissle 1917 (EcN) strain-mediated cancer therapy in a mouse tumor model. Here we demonstrate that gut microbiome modulation can be beneficial for colonization of EcN in tumor tissues and lead to improved survival of tumor-bearing mice treated with EcN.

**Abstract:**

In the past, different bacterial species have been tested for cancer therapy in preclinical and clinical studies. The success of bacterial cancer therapy is mainly dependent on the ability of the utilized bacteria to overcome the host immune defense system to colonize the tumors and to initiate tumor-specific immunity. In recent years, several groups have demonstrated that the gut microbiome plays an important role of modulation of the host immune response and has an impact on therapeutic responses in murine models and in cohorts of human cancer patients. Here we analyzed the impact of the gut microbiome on tumor colonization and tumor therapy by the *Escherichia coli* Nissle 1917 (EcN) strain. This EcN strain is a promising cancer therapy candidate with probiotic properties. In our study, we observed significantly better tumor colonization by EcN after antibiotic-induced temporal depletion of the gut microbiome and after two intranasal applications of the EcN derivate (EcN/pMUT-gfp Kn^r^) in 4T1 tumor-bearing syngeneic BALB/c mice. In addition, we demonstrated significant reduction in tumor growth and extended survival of the EcN-treated mice in contrast to phosphate-buffered saline (PBS)-treated tumor-bearing control animals. Multispectral imaging of immune cells revealed that depletion of the gut microbiome led to significantly lower infiltration of cytotoxic and helper T cells (CD4 and CD8 cells) in PBS tumors of mice pretreated with antibiotics in comparison with antibiotic untreated PBS—or EcN treated mice. These findings may help in the future advancement of cancer treatment strategies using *E. coli* Nissle 1917.

## 1. Introduction

Several bacteria, including *Salmonella* spp. [1], *Bifidobacterium longum* [2], *Eschericha coli* [3], *Listeria monocytogenes* [4] and *Clostridium* spp. [5,6] have been tested as anticancer agents in preclinical studies [7,8,9]. Most of these tumor-targeting bacteria were genetically modified to increase the effectiveness of cancer therapy [9,10]. Unfortunately, only a few bacterial strains have been successfully tested in human clinical trials with cancer patients. At this point, a number of Listeria vaccine strains have shown the best outcomes in cancer therapy and are currently in phase II and III clinical trials [9,11,12].

One of the new, promising candidates for bacterium-mediated cancer therapy is the *Escherichia coli* Nissle 1917 (EcN) strain, which has probiotic properties. This strain was isolated by Alfred Nissle in 1917 from a German soldier who remained healthy while his comrades succumbed to infections caused by *Shigella* [13]. This non-pathogenic EcN is the basis for an edible drug called “Mutaflor”^®^ (Ardeypharm, Germany), which is utilized for the treatment and prevention of gastrointestinal disorders, including ulcerative colitis [14], chronic constipation [15], Crohn’s disease [16] and irritable bowel syndrome [17]. Several engineered variants of EcN for the treatment of metabolic disorders [18], infectious diseases [19] and cancers [20] are currently in Phase I clinical trials. In addition, it was shown that the Nissle strain has immunomodulatory effects; for example, it suppresses immune-mediated damage and upregulates beneficial responses [21]. Furthermore, our group as well as others have previously demonstrated the ability of EcN to preferentially colonize tumor tissues [22,23,24,25,26].

In this study, we analyzed the impact of the gut microbiome on the antitumor capacity of an EcN derivate strain (EcN/pMUT-*gfp* Kn^r^) [27] in 4T1 syngeneic tumor-bearing BALB/c mice. Originating from a spontaneous mammary tumor in a BALB/c mouse [28], 4T1 is one of the most commonly used syngeneic tumor models for testing novel anticancer agents.

The gut microbiome is a collection of diverse microorganisms, such as bacteria, fungi, and archaea, which normally reside within the gastrointestinal tract [29,30]. Very recently, several research groups noted a relationship between the gut microbiome and cancer therapy [31,32]. As a result, the possible role of the gut microbiota in cancer treatment is currently under an intensive investigation.

Our findings demonstrate that antibiotic-induced, temporal depletion of the gut microbiome followed by intranasally applied bacterial treatment in 4T1 tumor-bearing BALB/c mice leads to increased tumor and gut colonization with EcN/pMUT-*gfp* Kn^r^ as well as to significant reduction in tumor growth compared to phosphate-buffered saline (PBS)-treated tumor-bearing control animals. In addition, this microbiome depletion leads to significantly lower infiltration of cytotoxic and helper T cells (CD4 and CD8 cells), as well as dendritic cells in tumors of antibiotic-pretreated 4T1 tumor-bearing mice in comparison with antibiotic untreated and/or EcN/pMUT-*gfp* Kn^r^ injected mice. These immunological studies were carried out with the help of CODEX-based multiplexed immunohistochemistry analysis [33,34], which allowed us localize over 10 surface markers in one tumor tissue section. These findings may become very important for the design of new approaches that involve modulation of the gut microbiome in order to improve cancer therapy with EcN.

## 2. Materials and Methods

### 2.1. Bacterium and Mammalian Cell Cultures

The bacterial strain EcN/pMUT-gfp Kn^r^ [27] is a derivate of *E. coli* Nissle 1917 (Ardeypharm GmbH, Herdecke, Germany) carrying a kanamycin (Kn)-resistance cassette as well as a green fluorescent protein (gfp) gene. In these experimental settings, the strain EcN/pMUT-gfp Kn^r^ was grown in Luria Bertani (LB) Broth medium (Sigma-Aldrich-L3022, Schnelldorf, Germany) containing 30 µg/mL kanamycin (Sigma-Aldrich, Schnelldorf, Germany). Bacteria were harvested and washed with endotoxin-free, sterile PBS (Dulbecco, Sigma-Aldrich, Schnelldorf, Germany, cat. No.: TMS-012-A) prior to injection.

The 4T1 mammary cancer cells (ATCC: CRL-2539) were cultured in DMEM medium (Thermo Fisher Scientific, Schnelldorf, Germany, 11965092) supplemented with 10% fetal bovine serum (FBS; Sigma- Aldrich, Schnelldorf, Germany, F4135) and 1% penicillin-streptomycin antibiotic solution (Sigma-Aldrich, Schnelldorf, Germany, P4333) at 37 °C in a humidified incubator with 5% CO_2_.

### 2.2. PCR and Western Blot Analyses of the Bacterial Strain EcN/pMUT-gfp Kn^r^

#### 2.2.1. PCR Analysis

The prior identity of the bacterial strain EcN/pMUT-gfp Kn^r^ was determined by PCR analysis with EcN-specific primers, as already described [13,27]. For this purpose, 100 µL overnight cultures of EcN/pMUT-gfp Kn^r^ and wild-type EcN (control) were boiled for 10 min at 100 °C and 1 µL of each culture was used as a template for PCR reactions.

#### 2.2.2. Western Blot Analysis

The strain EcN/pMUT-gfp Kn^r^ or the wild-type EcN (control) were grown in LB Broth medium (Sigma-Aldrich-L3022, Schnelldorf, Germany) with or without kanamycin. At different time points, the optical density of the cultures was measured at 600 nm with a BioPhotometer 6131 Spectrometer (Eppendorf, Germany). For detection of green fluorescent protein (GFP), 100 µL cultures of both strains were harvested and resuspended in sodium dodecyl sulfate (SDS) sample buffer. Samples were separated by 10% SDS-Polyacrylamide gel electrophoresis and subsequently transferred onto a nitrocellulose membrane (Whatman GmbH, Dassel, Germany). After blocking in 5% Bovine Serum Albumin (BSA: Albumin Fraktion V, Article no. 8076.4; Carl Roth, Karlsruhe, Germany) in 1x Tris-Buffered Saline (TBS; Trizma base, Article no. T1503; Sigma-Aldrich, Steinheim, Germany and sodium chloride, Article no. 73575; Fluka Analytical, Munich, Germany), the membrane was incubated with rabbit polyclonal antibody against green fluorescent protein (Santa Cruz Biotechnology, Heidelberg, Germany, sc-8334), The primary antibodies were detected using horseradish peroxidase-conjugated anti-rabbit (Abcam, Cambridge, UK, ab6721) secondary antibody, followed by enhanced chemiluminescence detection.

### 2.3. Antibiotic-Induced Microbiome Depletion in Mice

For gut microbiome depletion, mice received a cocktail of four antibiotics and one antimycotic [ampicillin 1 g/L (Sigma-Aldrich, Schnelldorf, Germany, A9518), vancomycin 0.35 g/L (Sigma-Aldrich, Schnelldorf, Germany, V2002), neomycin 1 g/L (Sigma-Aldrich, Germany, N1876), metronidazole 1 g/L (Sigma-Aldrich, Schnelldorf, Germany, M1547) and amphotericin B 0.1 g/L (Sigma-Aldrich, Schnelldorf, Germany, A2942)] in their drinking water, as described in previous reports [35,36]. Amphotericin B was added to prevent fungal overgrowth. The antibiotic cocktail was administered to BALB/c mice for a period of 7 days and was replaced every other day. At days 4 and 7 after antibiotic treatment (ABT), fecal samples of antibiotic-treated mice were collected and 100 mg of fecal samples (8–12 feces) were mechanically homogenized in 1 ml sterile PBS. The fecal suspensions were then plated on LB agar (Sigma-Aldrich, Schnelldorf, Germany, L3022), Schaedler agar with sheep blood plus hemin and vitamin K (Thermo Scientific Oxoid, Wesel, Germany, PB5034A) and Sabouraud agar (Roth, Germany, AE22.1) in doubles with 100 µL suspension on each plate. LB and Schaedler agar plates were incubated at 37 °C for 48 h, whereas Sabouraud agar plates were incubated at 30 °C for 72 h. The Schaedler agar plates were stored under anaerobic conditions by use of the Anaerocult A reagent (Merck kGaA, Darmstadt, Germany, Cat. No. 1.32381.0001) according to the manufacturer’s instructions.

### 2.4. Animal Experiments

Animal experiments were carried out in accordance with the protocol approved by the Government of Upper Franconia, Germany, according to the guidelines for the welfare and use of animals in cancer research (application No.: RUF-55.2.2.-2532-2-849).

The tumors were generated by implanting 1 × 10^5^ mammary carcinoma 4T1 cells subcutaneously into the right dorsal flank regions of 5- to 6-week-old female BALB/c mice (Charles River, Sulzfeld, Germany). After tumor cell implantation, the mice were randomized and divided into four groups. Tumor growth was determined with a digital caliper at least two times per week. Tumor volume was calculated by use of the modified ellipsoid formula [(length × width2)/2]. When tumors reached 100–250 mm^3^, the mice (*n*  =  5 mice per group) were injected intranasally (i.n.) twice, either with EcN/pMUT-gfp Kn^r^ (1 × 10^7^ CFU/15 µL PBS/ mice) or with PBS (control, 15 µL PBS/ mice) for a three-day interval. During this time, mice were monitored for changes in body weight as well as signs of toxicity. Mice that developed tumors with volumes greater than 1500 mm^3^ were euthanized as requested by the animal protocol.

### 2.5. Analysis of Bacterial Colony Forming Units (CFU) in Feces and Tumor Samples

Fecal samples were collected for 18 days after EcN/pMUT-gfp Kn^r^ injection. At different time points, about 8–12 feces (~100 mg) were mechanically homogenized in 1 mL sterile PBS. The fecal suspensions were then plated on LB agar plates supplemented with 30 µg/mL kanamycin (Sigma-Aldrich, Schnelldorf, Germany, B5264) and analyzed for EcN/pMUT-gfp Kn^r^ presence.

To determine bacterial load in tumor tissues, mice were euthanized and the tumors were excised, weighed and homogenized in sterile PBS. The homogenates were serially diluted and plated on LB agar plates containing 30 µg/mL kanamycin (Sigma-Aldrich, Schnelldorf, Germany, B5264). Resultant colonies were counted and the bacterial numbers were calculated as CFU per 1 g tumor tissue.

### 2.6. Multiplexed Immunohistochemistry (CODEX) of Tumor Tissue Sections

For histological studies of different treated 4T1 tumor-bearing syngeneic BALB/c mice, tumors were excised, snap-frozen in liquid nitrogen and embedded in Optimal Cutting Temperature compound (Sakura Finetek Europe, Netherlands). Tissue samples were sectioned (7 µm thickness) with the cryostat CM3050 S (Leica Microsystems GmbH, Wetzlar, Germany). The tumor samples were first fixed and labeled/stained using anti-mouse CODEX barcode-conjugated antibodies (Table 1) and analyzed by the CODEX system (Akoya Biosciences, Inc., Marlborough, MA, USA) according to the manufacturer’s protocol (CODEX User Manual, Chapter 5, page 42).

The fluorescence-labeled tissue samples were imaged using a BZ-X800 fluorescence microscope (Keyence, Osaka, Japan), equipped with the BZ-X800 Analyzer software (Keyence, Osaka, Japan). Finally, the raw data were processed using the CODEX processor software (AKOYA Bioscience, Marlborough, MA, USA), and analyzed with the QuPath vo. 3.2 software (https://qupath.github.io/, accessed on 15 July 2022. The statistical analysis was done using Microsoft Excel software.

### 2.7. Statistical Analysis

The significance of the results was calculated by Student’s t-test, and the Kaplan–Meier and log-rank (Mantel-Cox) tests. The comparison of CFUs between different treated groups was statistically evaluated in R with the function “wilcox.test” (https://www.r-project.org/, accessed on 6 July 2021). Results are displayed as means ± standard deviation (SD), *p* values of *<0*.05 were considered statistically significant. Asterisks indicate a significant difference between experimental groups (* indicates *p* < 0.05; ** indicates *p* < 0.01; *** indicates *p* < 0.001).

## 3. Results

### 3.1. Effects of Antibiotic-Induced Gut Microbiome Depletion and Intranasal (i.n.) EcN/pMUT-gfp Kn^r^ Applications on Tumor Growth in 4T1 /BALB/c Syngeneic Mouse Model

**Figure 1:**
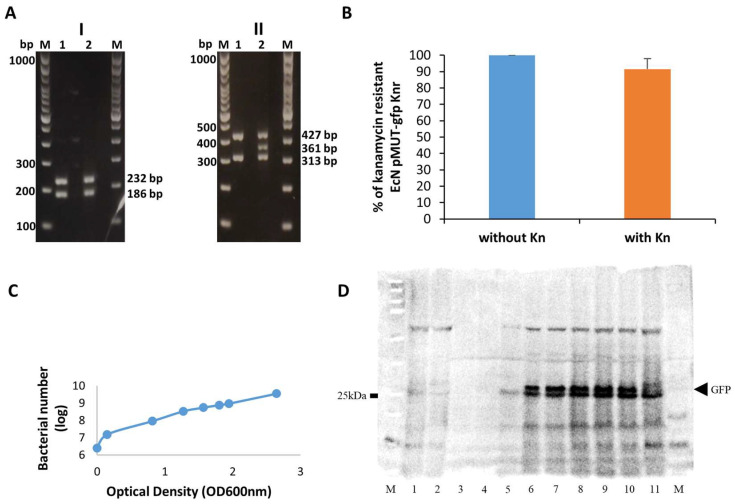
(**A**) PCR analysis of two different EcN strains. Chromosomal DNA specific (I) and plasmid specific (II) PCR analyses; Lanes: lane M: Marker (GeneRuler 100 bp DNA Ladder, Thermo Fisher Scientific, Germany, Cat. No. SM0321), lane 1: EcN/pMUT-gfp Kn^r^, lane 2: EcN (wild-type), (**B**) Analysis of plasmid stability of EcN/pMUT-gfp Kn^r^ bacteria after 20 generations with and without kanamycin (Kn) selection. The Kn positive cells of EcN/pMUT-gfp Kn^r^ bacteria were presented as mean values of two independent experiments. ± standard deviations in percentages. (**C**) Growth curve of EcN pMUT-gfp strain. At different time points, the optical density of the bacterial culture was measured at 600 nm (OD600) and the bacterial number was presented as a logarithm value (log). The measuring time points are represented as dots (**D**) Western blot analysis of cultures of EcN or EcN/pMUT-gfp Kn^r^ bacteria at different time points. Lane M: protein marker (Precision Plus Protein™ Kaleidoscop, Bio-Rad, Germany, Cat.No.1610375), lane 1: EcN (t = 120 min; OD_600_ = 0.16), lane 2: EcN (t = overnight; OD_600_ = 2.54), lane 3: EcN/pMUT-gfp Kn^r^, (t = 0 min; OD_600_ = 0.001), lane 4: EcN/pMUT-gfp Kn^r^ (t = 60 min; OD_600_ = 0.017), lane 5: EcN/pMUT-gfp Kn^r^ (t = 120 min; OD_600_ = 0.145), lane 6: EcN/pMUT-gfp Kn^r^ (t = 180 min; OD_600_ = 0.813), lane 7: EcN/pMUT-gfp Kn^r^ (t = 220 min; OD_600_ = 1.271), lane 8: EcN/pMUT-gfp Kn^r^ (t = 260 min; OD_600_ = 1.564), lane 9: EcN/pMUT-gfp Kn^r^ (t = 300 min; OD_600_ = 1.803), lane 10: EcN/pMUT-gfp Kn^r^ (t = 330 min; OD_600_ = 1.943), lane 11: EcN/pMUT-gfp Kn^r^ (t = overnight; OD_600_ = 2.646), lane M. Notes: t = 0, start of bacterial growth after inoculation. The GFP protein has a molecular weight of ~27 kDa. The results show a double band around 25 to 27 kDa.

*E. coli* Nissle 1917 (EcN) carries two naturally occurring plasmids pMUT1 and pMUT2, which have no known function, but both plasmids are stable within these bacteria [37]. For the construction of EcN/pMUT-gfp Kn^r^, the EcN strain was first cured of both plasmids pMUT1 and pMUT2 and then transformed with plasmid DNA pMUT2 carrying a Kn-resistance cassette as well as the gfp gene under the control of a constitutive promoter [27]. In the first step of our study, we validated the identity and the plasmid stability of the EcN/pMUT-gfp Kn^r^ strain in cultures grown with and without kanamycin selection by PCR and resistance to kanamycin (Figure 1A,B). The PCR analyses with EcN-specific primers (Table 2) showed the expected plasmid and chromosomal EcN patterns and the absence of the plasmid pMUT1 in the EcN/pMUT-gfp Kn^r^ strain compared to the wild-type EcN (Figure 1AII, line 1 vs. line 2). In addition, the plasmid pMUT-gfp Kn^r^ was stable in both overnight EcN/pMUT-gfp Kn^r^ cultures grown with and without kanamycin. Our analyses also demonstrated that less than 10% of EcN/pMUT-gfp Kn^r^ bacteria lost kanamycin resistance after 20 generations without antibiotic selection (Figure 1B).

Moreover, our Western blot analysis shows that the GFP protein expression in cultures of the EcN/pMUT-gfp Kn^r^ strain was stable at different time points (Figure 1C,D, lines 3–11).

The stable GFP expression and kanamycin resistance do facilitate the detection of EcN/pMUT-gfp Kn^r^ bacteria in mice.

For gut microbiome depletion, 5- to 6-week-old female BALB/c mice (Charles River, Sulzfeld, Germany) were treated with an antibiotic cocktail, as described in the Materials and Methods (Section 2.3). During this period, all animals were monitored daily for signs of toxicity. In addition, to confirm the efficacy of the antibiotic treatment (ABT), the presence of microorganisms in feces was determined at days 0, 4 and 7 after ABT (Table 3).

Our data demonstrated that under these experimental conditions, a significant reduction in numbers of bacteria and fungi was found after 4 days of the initial antibiotic treatment (Table 3).

**Figure 2:**
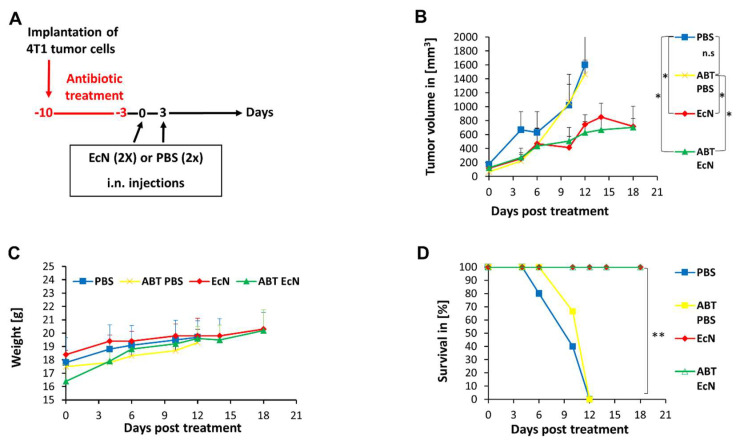
(**A**) Schedule of tumor implantation, antibiotic-induced microbiome depletion and EcN/pMUT-gfp Kn^r^ (EcN)-mediated tumor therapy in 4T1 tumor-bearing BALB/c mice. (**B**) Effects of intranasal bacterium or PBS injections on tumor growth of 4T1 tumor-bearing BALB/c mice with and without pretreatment with antibiotic (ABT). **PBS**—PBS-injected group; **ABT PBS**—antibiotic-pretreated and PBS-injected group; **EcN**—EcN/pMUT-gfp Kn^r^ injected group; **ABT EcN**—antibiotic-pretreated and EcN/pMUT-gfp Kn^r^ injected group. Results are displayed as means +/− standard deviation. The statistical significance was calculated by the Student’s *t*-test where * indicates *p* < 0.05 and “ns” indicates not significant. (**C**) Effect of different treatments on weight of 4T1 tumor-bearing BALB/c mice. (**D**) Survival of 4T1 tumor-bearing BALB/c mice after intranasal EcN/pMUT-gfp Kn^r^ or PBS treatment. The comparison of the survival between the three different treated groups was statistically evaluated by Kaplan–Meier and log-rank (Mantel-Cox) tests (GraphPad Prism, San Diego, CA). *p* < 0.05 was considered statistically significant. ** indicates *p* < 0.01.

Further, groups of BALB/c mice were subcutaneously injected with 1 × 10^5^ mammary carcinoma 4T1 cells into the right dorsal flank regions. On day 10 after implantation of the 4T1 cells, the tumors reached volumes between 100 and 250 mm^3^. Four groups of mice, two antibiotic-treated and two untreated, were injected intranasally (i.n.) either with EcN/pMUT-gfp Kn^r^ (1 × 10^7^ CFU/15 µL PBS/mouse) or with PBS only (15 µL PBS per mouse) (Figure 2A). After three days, all injections were repeated. In order to avoid possible complications resulting from the oral antibiotic treatment and the subsequent lysis effects of microorganism within the gastrointestinal tract, we preferred a nasal application of EcN/pMUT-gfp Kn^r^ to an oral application in these experimental settings. As shown in Figure 2B, the EcN/pMUT-gfp Kn^r^ treatment led to a statistically significant inhibition of tumor growth at day 12 post-injection in the two bacterium-treated groups compared to both PBS control groups. Due to an excessive tumor burden (>1500 mm^3^), all animals of the PBS groups were euthanized at this time point, as outlined in the animal protocol. During treatment, there was no significant difference in tumor volume reduction between the two EcN/pMUT-gfp Kn^r^-treated groups (i.e., antibiotic-pretreated vs. untreated).

Further, the toxicity of the bacterial therapy and the survival efficiency were analyzed by monitoring the tumor volume and the weight of mice (Figure 2C,D). With the exception of the first three days of treatment, all four mice groups showed similar mean weights over the course of this study (Figure 2C). There were no signs of bacterial or antibiotic-mediated distress. All EcN-treated mice (i.e., antibiotic-treated and untreated) displayed significantly enhanced survival, compared to PBS-treated animals (Figure 2D). Moreover, no mice from bacterium-treated groups developed tumors with volumes greater than the maximum allowable volume (1500 mm^3^) at 18 days post-bacterium injection (dpbi). Therefore, we terminated the animal experiments at this time point in order to analyze the presence of EcN bacteria in mice and investigate the possible role of host immune responses.

### 3.2. Distribution of EcN/pMUT-gfp Kn^r^ in Syngenic 4T1 Tumor Bearing BALB/c Mice

#### 3.2.1. Distribution of EcN/pMUT-gfp Kn^r^ Strain in Tumors

In order to study the effects of antibiotic-induced gut microbiome depletion, we first compared bacterial colonization in the tumor tissues of mice without and with antibiotic pretreatment (ABT) at the last time point of EcN/pMUT-gfp Kn^r^ treatment (Table 4).

The four highest bacterial titers (EcN/pMUT-gfp Kn^r^) were identified in tumors of the antibiotic-pretreated mice (Table 4, Group 1; ABT/EcN). On average, we detected about 50-fold higher CFUs in tumors of antibiotic-pretreated mice compared to no treatment (Table 4). Interestingly, no bacteria were found in three of five tumors of the untreated mice group at 18 dpbi. Moreover, antibiotic-pretreated, EcN-treated mice displayed significantly higher CFUs in tumors compared to EcN-treated animals only (ABT EcN/pMUT-gfp Kn^r^ vs. EcN/pMUT-gfp Kn^r^, *p* = 0.03445364; date accessed: 6 July 2021). The comparison of CFUs between these two different treatment groups was statistically evaluated in R (R Core Team) [38] with the function “wilcox.test”. *p* < 0.05 was considered statistically significant.

It seems to be that antibiotic pretreatment may prevent a rapid elimination of EcN from tumors and/or allow better bacterial delivery in tumor tissues. Taken together, our data clearly demonstrate that antibiotic-induced microbiome depletion has a positive effect on EcN/pMUT-gfp Kn^r^ replication and survival in tumor tissues.

#### 3.2.2. Presence of EcN/pMUT-gfp Kn^r^ in Fecal Samples of Mice with Intranasal Bacterium Injections

The presence and persistence of EcN/pMUT-gfp Kn^r^ in fecal samples of 4T1 tumor-bearing mice was analyzed at different days during tumor treatment (Table 5).

Our analysis demonstrated that EcN/pMUT-gfp Kn^r^ bacteria were still present in the feces of intranasally treated mice 18 days after bacterium injections. In the group of antibiotic-pretreated mice, we detected 10^2^−10^3^ CFU of EcN/pMUT-gfp Kn^r^ bacteria per 100 mg feces in the time interval between 11 and 18 dpbi. Although both groups of mice received similar bacterial doses, in the case of non-antibiotic-pretreated mice, low numbers of EcN/pMUT-gfp Kn^r^ (10^2^ CFU/100 mg of feces) were detected only at day 11 after bacterial injections.

Our data revealed that a temporal microbiome depletion leads to longer survival of the intranasally applied EcN/pMUT-gfp Kn^r^ bacteria in the gut as noted by analyses of the feces.

### 3.3. Role of Host Immune System in the Antitumor Mechanism of EcN/pMUT-gfp Kn^r^ Treatment

Multiplexed immunohistochemistry of the tumor tissues of mice with and without antibiotic pretreatment (ABT) at the last time point of PBS only or EcN/pMUT-gfp Kn^r^ treatments.

**Figure 3:**
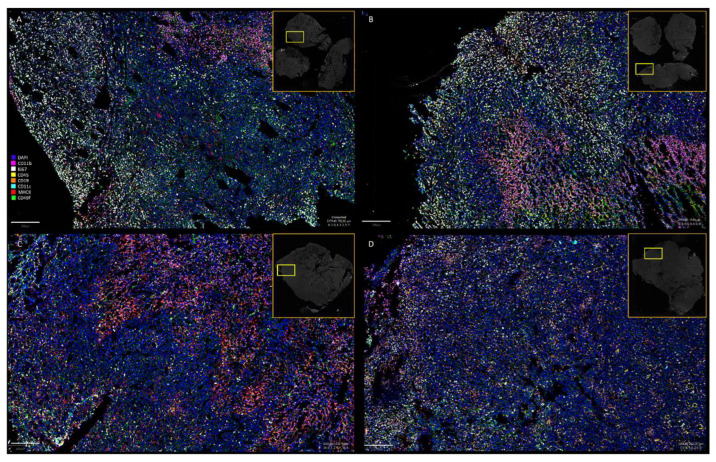
Multiplexed immunohistochemistry of tumor tissue sections of antibiotic-treated (ABT) and untreated mice after either bacteria (EcN) or PBS intranasal injections. The probes were stained with Ki67, CD11b, MHCII, CD19, CD11c, CD45 and CD49f antibodies and DAPI nuclear staining. (**A**): **PBS—**PBS-injected group; (**B**): **ABT PBS**—antibiotic-pretreated and PBS-injected group; (**C**): **EcN**—EcN/pMUT-gfp Kn^r^ injected group; (**D**): **ABT EcN**—antibiotic-pretreated and EcN/pMUT-gfp Kn^r^-injected group. All pictures are presented at the same magnification. The position of presented area of the section is indicated by yellow rectangles. Scale bars represent 200 µM.

**Figure 4:**
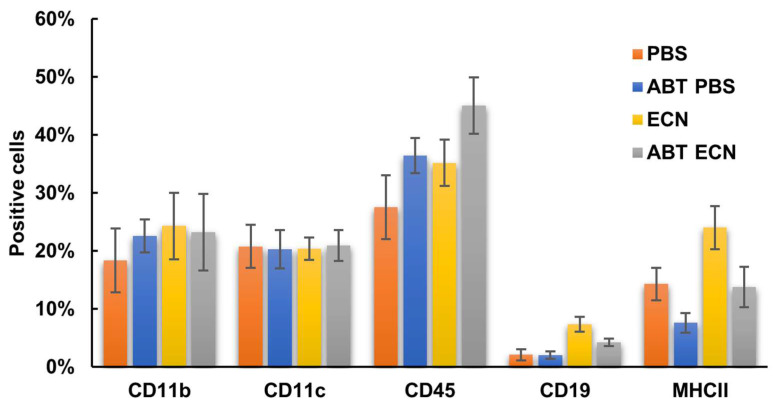
Percent [%] of CD11b-, CD11c-, CD45-, CD19- or MHCII-positive cells in tumor tissue prepared from antibiotic-treated (ABT) and untreated mice after either bacterial (ECN) or PBS injections. **PBS—**PBS-injected group; **ABT PBS**—antibiotic-pretreated and PBS-injected group; **EcN**—EcN/pMUT-gfp Kn^r^-injected group; **ABT EcN**—antibiotic-pretreated and EcN/pMUT-gfp Kn^r^-injected group.

To investigate the potential role of the host immune system, we analyzed the effects of antibiotic-induced gut microbiome depletion alone or together with intranasal bacterium injections on presence of host immune cells in the tumor tissue of 4T1-tumor-bearing mice. We used the CODEX multiplexed imaging technology that enables quantitative detection of at least 30 different markers at single-cell-level resolution [34]. Fresh frozen tissues (*n* = 3 for each time points) were stained with a panel of 10 antibodies (Ki67-, CD11b-, MHCII-, CD19-, CD4-, CD3-, CD8a-, CD11c-, CD45- and CD49f-antibody, Table 1) to identify major immune cell phenotypes including subsets of antigen presenting cells (mainly dendritic cells (DCs) and macrophages), B and T lymphocytes. We identified different immune cell densities and distribution after different treatment (Figure 3). However, there were no significant differences in proliferation (Ki67+), number of infiltrating leukocytes (CD45+) and B cells among the four tested groups (Figure 4).

**Figure 5:**
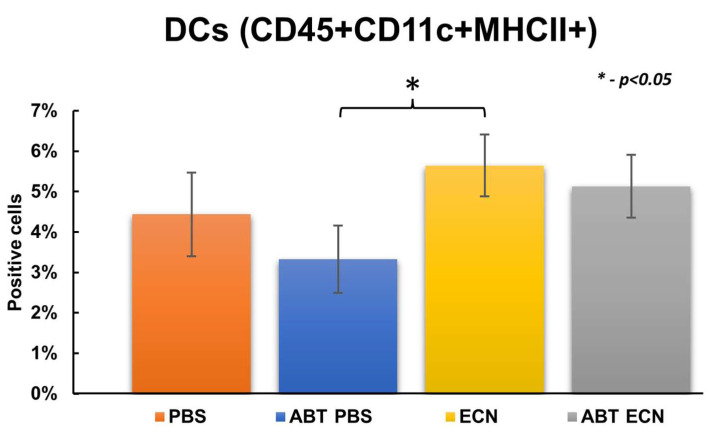
Percent [%] of dendritic cells (DCs) in tumor tissue prepared from antibiotic-treated (ABT) and untreated mice after either bacterial (ECN) or PBS treatments. PBS—PBS-injected group; ABT PBS—antibiotic-pretreated and PBS-injected group; EcN—EcN/pMUT-gfp Kn^r^-injected group; ABT EcN—antibiotic-pretreated and EcN/pMUT-gfp Kn^r^-injected group. Data are representative of three independent experiments (*n* = three mice per group). The statistical significance was calculated by the Student’s *t*-test where * indicates *p* < 0.05.

**Figure 6:**
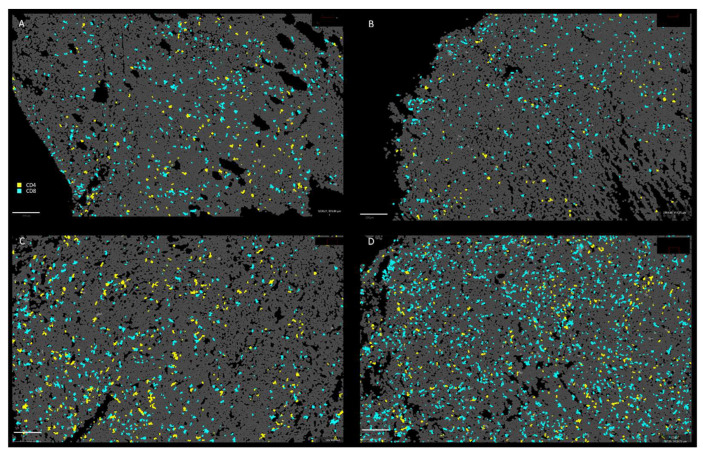
Tumor tissue sections prepared from antibiotic-treated (ABT) and untreated mice after either bacterial (EcN) or PBS injections stained with CD4 and CD8 antibodies and analyzed by CODEX multiplexed imaging. CD4+ and CD8+ cells in tumor tissue sections (presented areas correspond to areas in Figure 3). (**A**): **PBS—**PBS-injected group; (**B**): **ABT PBS**—antibiotic-pretreated and PBS-injected group; (**C**): **EcN**—EcN/pMUT-gfp Kn^r^ injected group; (**D**): **ABT EcN**—antibiotic-pretreated and EcN/pMUT-gfp Kn^r^ injected group. All pictures in this set were taken at the same magnification. Scale bars represent 200 µM.

**Figure 7:**
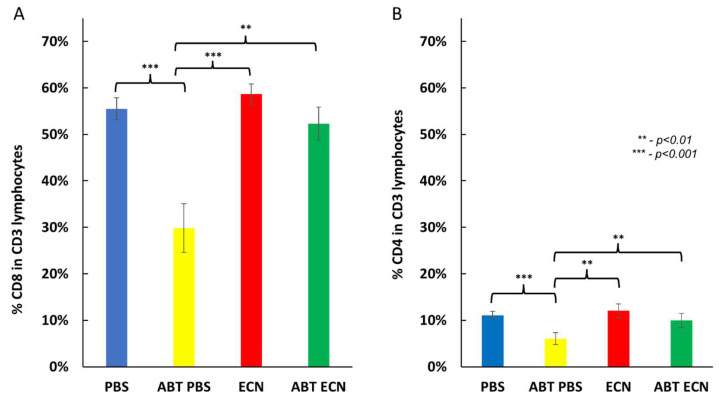
Identification of subsets of T lymphocytes. Different tumor tissue sections stained with CD3, CD4 and CD8 antibodies and were analyzed by CODEX multiplexed imaging. (**A**) Percent [%] of CD8+ cells in CD3+ cell population. (**B**) Percent [%] of CD4+cells in CD3+ cell population. **PBS—**PBS-injected group; **ABT PBS**—antibiotic-pretreated and PBS-injected group; **EcN**—EcN/pMUT-gfp Kn^r^ injected group; **ABT EcN**—antibiotic-pretreated and EcN/pMUT-gfp Kn^r^ injected group. Data are representative of three independent experiments (*n* = three mice per group). The statistical significance was calculated by the Student’s t-test where ** indicates *p* < 0.01 and *** *p* < 0.001.

In contrast, significantly lower infiltration of DCs (population of CD45, CD11c and MHCII positive cells) was found in the tumors of antibiotic-pretreated PBS mice in comparison with antibiotic-untreated mice after EcN injections. (Figure 5). In addition, significantly lower infiltration of cytotoxic and helper T cells (CD4 and CD8 cells) was also associated with the antibiotic pretreatment (ABT PBS mice), but not in response to EcN. (Figure 6 and Figure 7). In these analyses we used the combination of CD3, CD4 and CD8 antibodies for staining and identification of subsets of T lymphocytes (Figure 7). However, the mechanism of how the number of DCs and T cells in the tumor tissue may have influenced *E. coli* Nissle-mediated antitumor therapeutic efficacy is currently unknown.

## 4. Discussion

In this study, we investigated the impact of the gut microbiome on the anti-tumor activity of the *E. coli* Nissle 1917 derivative, EcN/pMUT-gfp Kn^r^, in a 4T1 tumor-bearing syngeneic BALB/c mouse model. In recent years, several studies have demonstrated that cancer therapy can be enhanced and improved by modulation of the gut microbiome [31,39,40]. The gut microbiome has an influence on several important host functions, which include nutrient acquisition, organ development and immune modulation [41,42]. Here we significantly reduced the gut microorganisms by antibiotic-induced microbiome depletion with an antibiotic cocktail consisting of four antibiotics and one antimycotic compound for a period of at least 7 days. Throughout the intranasally delivered bacterial therapy, we compared the antitumor effects of the EcN/pMUT-gfp Kn^r^ strain with or without antibiotic pretreatment. In our experimental setting, we preferred the intranasal route of bacterial application. In that way, we hoped to avoid possible complications through oral bacterial application and oral antibiotic treatment as well as subsequent effects of microorganism’s lysis within the gastrointestinal tract. In addition, it is known that the intranasal route of administration of EcN is more efficient than the oral route and leads to the persistence of these *E. coli* bacteria in the gut of healthy mice [43]. Our data showed that, after temporal antibiotic-induced depletion of the gut microbiome, the presence of EcN/pMUT-gfp Kn^r^ bacteria in tumor tissues was significantly higher (about 50-fold) than in tumors of antibiotic-untreated mice (Table 4). One possible reason for this finding may be that microbiome depletion modulates the immune system and probably affects nutrient acquisition in tumor-bearing mice. Stritzker et al. already reported that the depletion of macrophages results in elevated EcN colonization in tumor tissue, while bacterial strains defective for aromatic amino acid biosynthesis show increased tumor specificity [23]. Therefore, to be sure that the host immune system is still functional after microbiome depletion, we analyzed major immune cell phenotypes including subsets of antigen presentig cells (mainly dendritic cells and macrophages), B and T lymphocytes in the tumor tissue on the last day of EcN or PBS treatment by multiplexed immunohistochemistry. Compared to FACS analysis, the method does not require cell isolation that may result in variable cell loss and retains the tumor structure allowing for spatial analysis of tumor [44]. Our data revealed that antibiotic pretreatment led to strongly reduction on the number of microorganisms in gut (Table 3) and significantly lower infiltration of T cells in the tumors of ABT PBS-treated mice in comparison with PBS or EcN-treated groups (Figure 6 and Figure 7). Interestingly, intranasal applications of EcN in antibiotic-pretreated and untreated mice led to significantly increased numbers of CD3+CD4+ T-cells (possible Th1 T helper cells) and CD3+CD8+ (cytotoxic T cells) in comparison with ABT PBS-treated mice. This could be evidence that *E. coli* Nissle infection stimulates T cell infiltration in the tumor tissue. Moreover, a Th1 response was observed in mice after *E. coli* Nissle infection [45] and such a Th1 response leads to tumor growth inhibition in the 4T1 mouse tumor model [46]. In addition, significantly lower DCs infiltration was found in the tumors of antibiotic-pretreated PBS mice in comparison with antibiotic-untreated mice after EcN injections only. However, until now, the mechanism of *E. coli* Nissle-mediated antitumor effects has not been fully understood. It is possible that the presence of EcN in tumor tissue may elicit the host immune response against the tumor and/or induce changes in the tumor microenvironment—leading to antitumor effects [22,26,47]. In this study, EcN/pMUT-gfp Kn^r^ treatment resulted in a statistically significant inhibition of tumor growth compared to PBS controls, but did not result in complete tumor regressions (Figure 2B). However, our findings demonstrate that temporary depletion of the gut microbiome may have a positive effect on EcN tumor colonization and replication. Here, the microbiome depletion led to the significantly extended survival of EcN/pMUT-gfp Kn^r^ in the tumor tissue and longer persistence in gut (Table 4 and Table 5). The mechanism for this phenomenon is still unknown, but antibiotic-induced microbiome depletion had no direct effect on EcN-mediated antitumor efficacy under these experimental conditions. On the other hand, enhanced tumor colonization or longer persistence of EcN in the host may be beneficial for targeted cancer therapies and the delivery of antitumor drugs [10,48].

In general, the role of gut bacteria in modulating the anticancer response is a controversial topic [32]. However, it is known that several different gut bacteria positively influence tumor treatment, but no studies have identified the corresponding mechanism until now [49]. Interestingly, after depletion of the gut microbiome by antibiotics, both antitumoral and protumoral activity have been described [49]. In our study, we showed that after temporal antibiotic-induced depletion of the gut microbiome, EcN can stimulate host immune cells such as dendritic cells or Th1 responses, resulting in significantly prolonged survival of the EcN treated 4T1 tumor-bearing mice (Figure 2). Since the gut microbiome is a complex ecosystem, further research needs to be done to understand how the microbiome affects the host response to tumors and tumor-associated EcN bacteria.

Thus, the combination of EcN-mediated tumor therapy and microbiome modulation may be a fruitful, novel option for enhancing multiple forms of cancer therapy in general.

## 5. Conclusions

The significant incidence and mortality associated with cancers continue to challenge modern medicine to develop more reliable therapies. One of the most promising novel cancer therapies is bacterium-mediated cancer therapy. This method is mainly based on the capability of bacteria to preferentially target cancer tissue and to initiate tumor-specific immunity. Until now, several different bacterial species have been tested with convincing results in preclinical and clinical studies. In the current study, we demonstrated for the first time that microbiome modulation could be helpful for the extended survival of EcN/pMUT-gfp Kn^r^ in the tumor tissue and significantly improved survival of the EcN-treated, tumor-bearing mice. On the basis of these data, we propose that microbiome modulation may become a new option for enhancing bacterium-mediated cancer therapies.

## Figures and Tables

**Table 1:** List of the anti-mouse antibodies used.

Product Code	Product (Target—Dye-Reporter)	Vendor
4230019	**Ki67**(AKYP0052)-BX047—Atto 550	Akoya Biosciences, Inc.
4250016	**CD4**(AKYP0041)-BX026—Atto 550	Akoya Biosciences, Inc
4350014	**CD3**(17A2)-BX021—Cy5	Akoya Biosciences, Inc
4250014	**CD19**(AKYP0033)-BX020—Atto 550	Akoya Biosciences, Inc
4250017	**CD8a**(AKYP0044)-BX029—Atto 550	Akoya Biosciences, Inc
4250003	**MHC II**(AKYP0006)-BX014—Atto 550	Akoya Biosciences, Inc
4450015	**CD11b**(AKYP0040)-BX025—Alexa Fluor™ 750	Akoya Biosciences, Inc
4550108	**CD11c**(AKYP0045)-BX030—Cy5	Akoya Biosciences, Inc
4550102	**CD49f**(AKYP0018)-BX033—Cy5	Akoya Biosciences, Inc
4450002	**CD45**(AKYP0005)-BX007—Alexa Fluor™ 750	Akoya Biosciences, Inc

**Table 2:** List of PCR primers used.

Primers	Origin DNA	DNA Sequences	Length
4L2 4R2	Chromosomal	5′-GGG CGA TCG GAT TTA ATC AT-3′ 5′-CGA GGA CTC GGA GCT TAC TG-3′	186 bp
5L1 5R1	Chromosomal	5′-GCC TCT CGC AAC TTA ACG AC-3′ 5′-AGT TAT CCA GCG TTG CCA TC-3′	232 bp
Muta5 Muta6	Plasmid pMUT1	5′-AAC TGT GAA GCG ATG AAC CC-3′ 5′-GGA CTG TTC AGA GAG CTA TC-3′	361 bp
Muta7 Muta8	Plasmid pMUT2	5′-GAC CAA GCG ATA ACC GGA TG-3‘ 5′-GTG AGA TGA TGG CCA CGA TT-3′	427 bp
Muta9 Muta10	Plasmid pMUT2	5′-GCG AGG TAA CCT CGA ACA TG-3′ 5′-CGG CGT ATC GAT AAT TCA CG-3′	313 bp

bp: base pairs.

**Table 3:** Effect of antibiotic treatment (ABT) on the number of microorganisms per gram feces of BALB/c mice (*n* = 3) at different time points.

Time Point (Day)/CFU/g Feces	Day 0 before ABT	4 Days after ABT	7 Days after ABT
CFUs on LB agar plates	1.76 × 10^5^ ± 4.82 × 10^4^	3.19 × 10^4^ ± 3.05 × 10^3^	n.d.
CFUs on Sabouraud agar plates	3.27 × 10^3^ ± 8.16 × 10^2^	n.d.	n.d.
CFUs on Schaedler agar plates	2.62 × 10^7^ ± 3.78 × 10^6^	1.60 × 10^5^ ± 1.05 × 10^5^	n.d.

Two independent fecal samples of 8–12 feces (approximately 100 mg) were collected at different time points. The number of microorganisms were analyzed as described in materials and methods (Section 2.3.); n.d.: not detected.

**Table 4:** CFU of EcN/pMUT-gfp Kn^r^ (EcN) in tumor tissues of 4T1BALB/c mice at 18 dpbi.

Mouse No.	CFU /per 1g Tumor Tissue
Group 1/Antibiotics pretreated (ATB)/EcN	
206/ABT	9.64 × 10^2^ ± 2.14 × 10^2^
207/ABT	1.82 × 10^2^
208/ABT	7.37 × 10^3^ ± 3.39 × 10^2^
209/ABT	1.60 × 10^3^
210/ABT	6.2 × 10
Group 2/No antibiotics/EcN	
291	9.2 × 10
290	n.d.
289	n.d.
288	1.18 × 10^2^
287	n.d.

The data were determined by standard CFU assays on LB agar plates with 30 µg/mL kanamycin using aliquots of homogenized tumors. For each tumor, two aliquots of 100 µL were analyzed and the data were displayed as mean cfu ± standard deviation (SD) per 1 g tumor tissue. ABT—antibiotic-pretreated mouse; n.d.: not detected.

**Table 5:** Detectable CFU of EcN/pMUT-gfp Kn^r^ (EcN) bacteria in feces of 4T1 tumor-bearing mice.

Mice Group/ Day of Treatment	Group 1 ABT */EcN 11dpbi	Group1 ABT*/EcN 18dpbi	Group 2/EcN 11dpbi	Group 2/EcN 18dpbi
CFU per100 mg feces	1.8 × 10^3^ ± 4.0 × 10^2^	9.0 × 10^2^ ± 1.0 × 10^1^	1.0 × 10^2^	n.d.

Two independent fecal samples of 8–12 feces (approximately 100 mg) for each group of mice (n  =  5 mice per group), were homogenized in 1 mL sterile PBS at day 11 or day 18 post-bacterial injection (dpbi). Aliquots (100 µL) of each homogenized sample were plated on LB agar plates containing 30 µg/mL kanamycin. Resultant colonies were counted and the data displayed as mean cfu ± standard deviation (SD) per 100 mg feces. * ABT—antibiotic-pretreated mice group. n.d.: not detected.

## Data Availability

The data presented in this study are available on request from the corresponding authors.
